# Brewers’ Yeast as a Remedy

**Published:** 1889-08

**Authors:** 


### BREWERS' YEAST AS A REMEDY.

In olden times brewers' yeast was considered a tonicum and antisepticum, and often prescribed in enteric fevers. (S. L. has verified its benefit in typhoids in many a hard case, prescribing a tablespoonful of fresh brewers' yeast in a pint of water as a beverage, often in alternation with a phosphoric acid lemonade, and no other treatment necessary. This was in ye olden times, when

Homœopathy was not yet accepted.) The English physicians consider it a mild purgative. Mettenheimer gives it with success in obstinate constipation, and others found it of equal benefit in catarrhal and saburral diarrhœa, among the disturbing contents, and thus restoring normal digestion. In many cases of phthisis tuberculosus it checked the exhausting diarrhœa after the failure of other remedies. In fact, in the catarrhal affection of the apices pulmonum, that first stage of threatening consumption, it relieved the cough and the short breathing, and in some cases restored health, especially where the vital force had to fight against a tubercular disposition. Strict individualization is necessary, says Mettenheimer, as some patients can hardly bear a teaspoonful. Lay people often use brewers' yeast in hot milk for chronic constipation, and for ages its external use has been with them a favorable application in burns, and internally and externally in scrofulous skin diseases.

*Allg. Med. Centr., Zeit.* 36, 1889.

Have we here a new antipsoricum? In my practice it always holds a good place, and as a gargle from simple angina to true diphtheria, and it did me as much good as the alcohol gargle recommended by Granvogl. Has it ever been proved, or will such provings in different potencies bring out symptoms which may aid us in the cure of severe zymotic diseases? We cannot shut our eyes to the bacteriological studies of the present age, though I believe that Gregg's decomposed fibres and the bacteria are one and the same thing, effects not the cause of disease, and is it allowable to a strict follower of Hahnemann to use brewers' yeast for their destruction? S. L.

